# Structure-guided post-SELEX optimization of an ochratoxin A aptamer

**DOI:** 10.1093/nar/gkz336

**Published:** 2019-05-07

**Authors:** Guohua Xu, Jiajing Zhao, Na Liu, Minghui Yang, Qiang Zhao, Conggang Li, Maili Liu

**Affiliations:** 1 Key Laboratory of Magnetic Resonance in Biological Systems, State Key Laboratory of Magnetic Resonance and Atomic and Molecular Physics, National Center for Magnetic Resonance in Wuhan, Wuhan National Laboratory for Optoelectronics, Wuhan Institute of Physics and Mathematics, Chinese Academy of Sciences, Wuhan 430071, P.R. China; 2 University of Chinese Academy of Sciences, Beijing 100029, P.R. China; 3 State Key Laboratory of Environmental Chemistry and Ecotoxicology, Research Center for Eco-Environmental Sciences, Chinese Academy of Sciences, Beijing 100085, P.R. China

## Abstract

SELEX is the cornerstone for aptamer research with broad applications in biosensors and medicine. To improve the affinity of selected aptamers, we propose a structure-guided post-SELEX approach, an optimization method based on the precise secondary structure of the aptamer–ligand complex. We demonstrate this approach using the Ochratoxin A (OTA) aptamer. Guided by the structure, we designed a new aptamer whose affinity is improved by more than 50-fold. We also determined the high-resolution NMR structure of the new aptamer-OTA complex and elucidated the discriminatory recognition mechanism of one atomic difference between two analogs, OTA and OTB. The aptamer forms an unusual hairpin structure containing an intramolecular triple helix, which is not seen in the previously determined aptamer complex. The π–π stacking, the hydrophobic interaction, hydrogen bonds and halogen bonds between OTA and the aptamer contribute to the recognition of OTA, and the halogen bonds play an important role in discriminating between OTA and OTB. Our results demonstrate that the structure-guided post-SELEX approach improves aptamers affinity. An improved OTA biosensor system might be developed using this new strategy.

## INTRODUCTION

Aptamers are short single-stranded nucleic acid sequences usually generated *in vitro* using SELEX (Systematic Evolution of Ligands by EXponential enrichment) (1,2). Aptamers have great potential applications in biosensors, therapeutics and drug delivery due to their capability of binding specific ions, small molecules, proteins, sugars, lipids and even whole cells (3–14).

SELEX involves multi-round polymerase-chain-reaction (PCR) amplification of target-bound oligonucleotides. Sometimes, low-affinity oligonucleotides are selected because they amplify more efficiently than high-affinity oligonucleotides (15). In such cases, post-SELEX optimization can be applied to enhance affinity. Several post-SELEX optimization strategies including truncation, chemical modification and mutagenesis have been used (16). However, post-SELEX is not precise and usually requires several rounds of selection. Here, we propose a structure-guided post-SELEX approach: aptamer optimization based on the precise secondary structure of the aptamer–ligand complex. We demonstrate this approach using an Ochratoxin A (OTA) binding aptamer.

OTA, a naturally occurring mycotoxin produced by several fungal species including, *Aspergillus ochraceus* and *Penicillium verrucosum*, is one of the most widespread food contaminants. It is nephrotoxic and carcinogenic and poses a threat to both the health of humans and other animals (17,18). Aptamers that bind OTA were generated using SELEX in 2008 (19). Since then, methods for detecting and quantifying OTA using aptamers have been developed. OTA aptamers are among the top 10 and are especially useful in the food industry (14). However, there are no high-resolution 3D structures of OTA–aptamers complexes.

**Figure 1.**
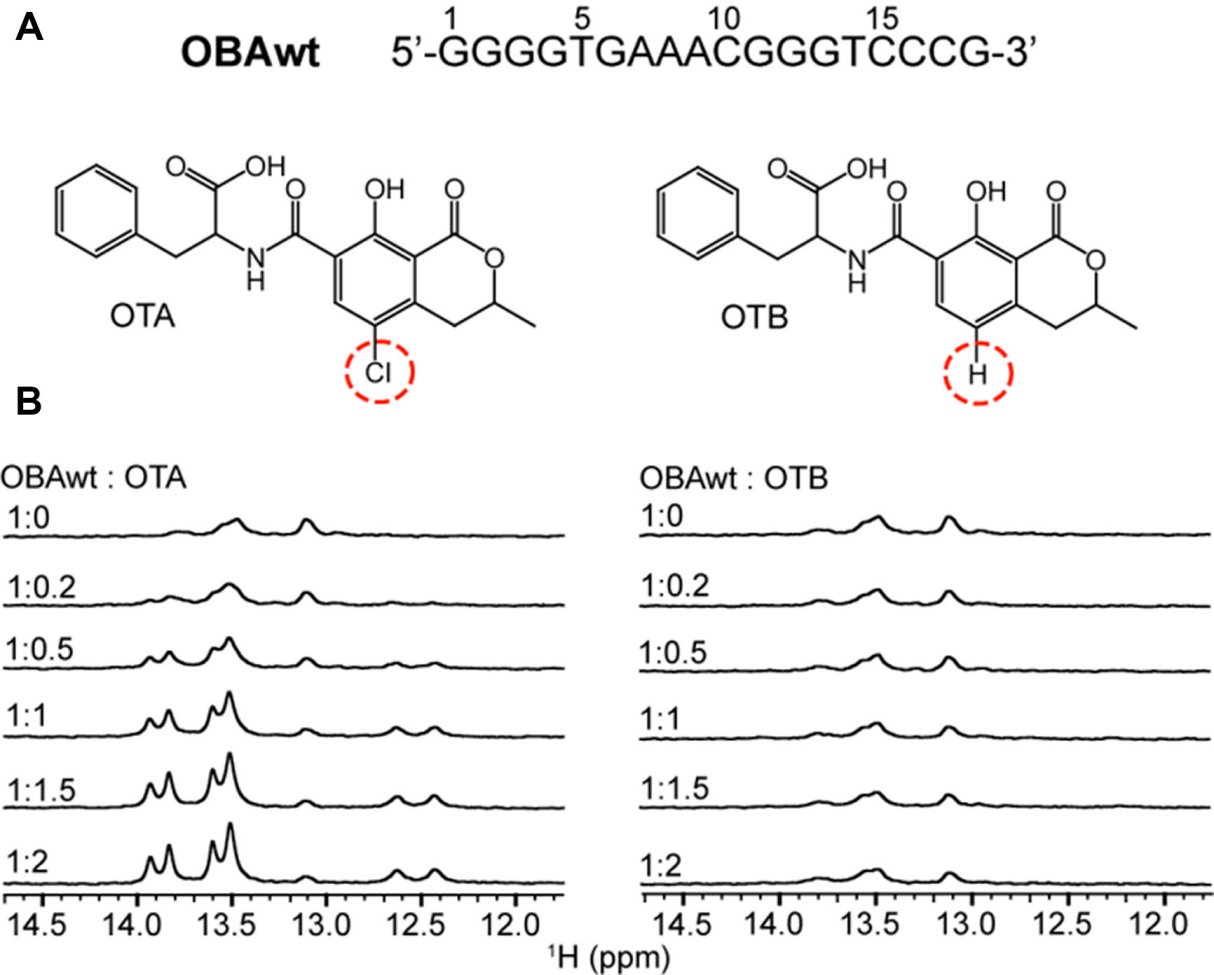
^1^H NMR spectra of the OBA aptamer titrated with OTA and OTB. (**A**) Sequence of aptamer OBAwt and chemical structures of OTA and OTB. (**B**) Imino regions of ^1^H NMR spectra of OBAwt (0.1 mM) titrated with OTA (left) and OTB (right) in the presence of 10 mM Mg^2+^ at 288 K.

The aptamer sequence selected by Cruz-Aguado *et al.* (19), GGGGTGAAACGGGTCCCG (OBAwt), binds OTA with a dissociation constant in the μM range. OBAwt does not bind Ochratoxin B (OTB), a structural analogue of OTA, even though there is only a one atom difference: the chlorine in the isocoumarin ring of OTA is a hydrogen in OTB (Figure 1). Structural determination of the aptamer–ligand complex is essential for understanding the molecular mechanism of aptamer recognition and for the rational design of highly functional aptamers. Having determined the structure of OTA with aptamers, we use a structure-guided post-SELEX approach to designed a new aptamer with a 50-fold affinity improvement. The new aptamer-OTA complex structure provides a detailed structural basis for understanding recognition.

## MATERIALS AND METHODS

### Sample preparation

Ochratoxins A and B were purchased from Sigma, and dissolved in d_6_-DMSO. Oligodeoxyribonucleotides were synthesized by Invitrogen (Shanghai, China) and Sangon Biotechnology Co., Ltd. (Shanghai, China). NMR samples were prepared by dissolving the lyophilized DNA powder in phosphate buffer, either in D_2_O or a 90%/10% H_2_O/D_2_O. The concentration of nucleic acid for NMR samples was 0.05–1.8 mM. The NMR buffer comprised 10 mM Na_2_HPO_4_/KH_2_PO_4_ (pH 7.4), 10 mM MgCl_2_.

### NMR

Spectra were recorded on Bruker 600, 700 and 850 MHz NMR spectrometers equipped with CyroProbes. ^1^H spectra were acquired with 128–1024 scans and a relaxation delay 2 s. TOCSY (20) and DQF-COSY (21,22) spectra were collected in D_2_O at 288 K. NOESY spectra with H_2_O suppression [Watergate W5 with gradients (23,24)] were collected in D_2_O at 288 K and 90%/10% H_2_O/D_2_O at 288 and 298 K, respectively, with mixing time of 100, 120 and 300 ms. These spectra comprised 2048 × 512 complex points and were acquired with a 2 s relaxation delay. JR-HMBC spectra (25) were acquired at 298 K with 8 K scans and comprised 2048 × 48 complex points. ^1^H chemical shifts were referenced to 2,2-dimethylsilapentane-5-sulfonic acid at 0 ppm.

^31^P NMR were collected at 288, 298 and 310 K and referenced to external 85% H_3_PO_4_. ^31^P–^1^H COSY (26) and HSQC (27) spectra were recorded at 298 and 310 K, respectively. ^31^P assignments were accomplished using a combination of ^1^H–^1^H NOESY, COSY, TOCSY and hetero-nuclear ^31^P–^1^H COSY data. Spectra were processed using Bruker TopSpin 3.2 and analyzed using SPARKY (28).

### Structure calculation

The OBA3─OTA structures were calculated from an extended unfolded ssDNA following standard Xplor protocols (29) using Xplor-NIH 2.47 (30,31). Restraints, including NOEs, sugar pucker, dihedral angle, and hydrogen bond were used. NOEs restraints were based on NOESY spectra with a mixing time 120 ms. Dihedral angles restraints were used to restrict the glycosidic dihedral angles (χ) and the sugar backbone dihedral angles β, γ and ϵ. Based on intraresidue H1′-H6/8 cross-peak intensities, the χ angles of all other residues were restricted to the *anti*-configuration, except for T15 whose χ angle was restrained to the *syn*-configuration. Based on the *J*-coupling constants of ^31^P(*n*)─H5′/H5″(*n*) and H3′ (*n* – 1)─^31^P(*n*) obtained from ^31^P─^1^H COSY (Supplementary Figure S6) and HSQC experiments with various *J*-couplings, the β angles were restrained to 180° ±60° for residues G2, G4, G5─C11, G13─G19 and 60° ±40° for G12. The ϵ angles were restrained to 240 ± 145° for G2, G5─C11, G13─T15 and 60 ±40° for G12, C16 and C17. The γ angles were restrained to 50 ±30° for G2─C6, A8, G10─C11, G13─G14, C16─G19 and 180 ± 40° for T15, based on the intensities of H3′–H5′/H5″ and H4′–H5′/H5″ cross-peaks in NOESY spectra (32). Sugar puckering conformations were restricted based on COSY spectra where the intensities of cross-peaks depend directly on the magnitudes of the coupling constants (33). Repulsive restraints were applied to several pairs of well-resolved protons, which did not give NOEs. The ten lowest energy conformations were chosen for further analysis. Structures were analyzed using MOLMOL (34) and displayed using the PyMOL Molecular Graphics System, Version 0.99rc6, Schrödinger, LLC.

### Fluorescence polarization

Polarization was measured using a Horiba FluoroMax-4 spectrofluorometer at room temperature. Slits for the excitation and the emission were set at 5 nm. Polarization was measured with excitation at 375 nm and emission at 440 nm. Three data points were collected and averaged. The binding buffer was the same as the NMR buffer. The concentration of OTA was 500 nM in a final volume 400 μl. Polarization was measured as the aptamer was added to the OTA solution. *K*_d_ was determined as described (35).

## RESULTS

### OBA aptamer binds OTA but not OTB

OTA and OTB, differ by one atom: OTB lacks the chlorine atom in the isocoumarin ring (Figure 1A). The ^1^H chemical shifts of imino groups in DNA are sensitive to structure and were employed to detect the interaction of aptamers with OTA and OTB. Figure 1B shows the imino region of the ^1^H NMR spectra of aptamer OBAwt titrated with OTA and OTB in the presence of Mg^2+^. In the absence of OTA, OBAwt shows several broad resonances, indicating that OBAwt alone lacks defined structure. With the addition of OTA, several resonances appear and the linewidths decrease, indicating OTA binding. When the OTA:OBAwt mole ratio is larger than one, there is no change in ^1^H NMR spectra of imino groups with increasing OTA concentration, suggesting a 1:1 stoichiometry. Unlike OTA, ^1^H NMR resonances from imino groups do not change with the addition of OTB, indicating that OBAwt does not bind OTB. These titration experiments demonstrate that OBAwt specifically binds OTA and can discriminate the single atom difference.

### Structure of the OBAwt─OTA complex

**Figure 2.**
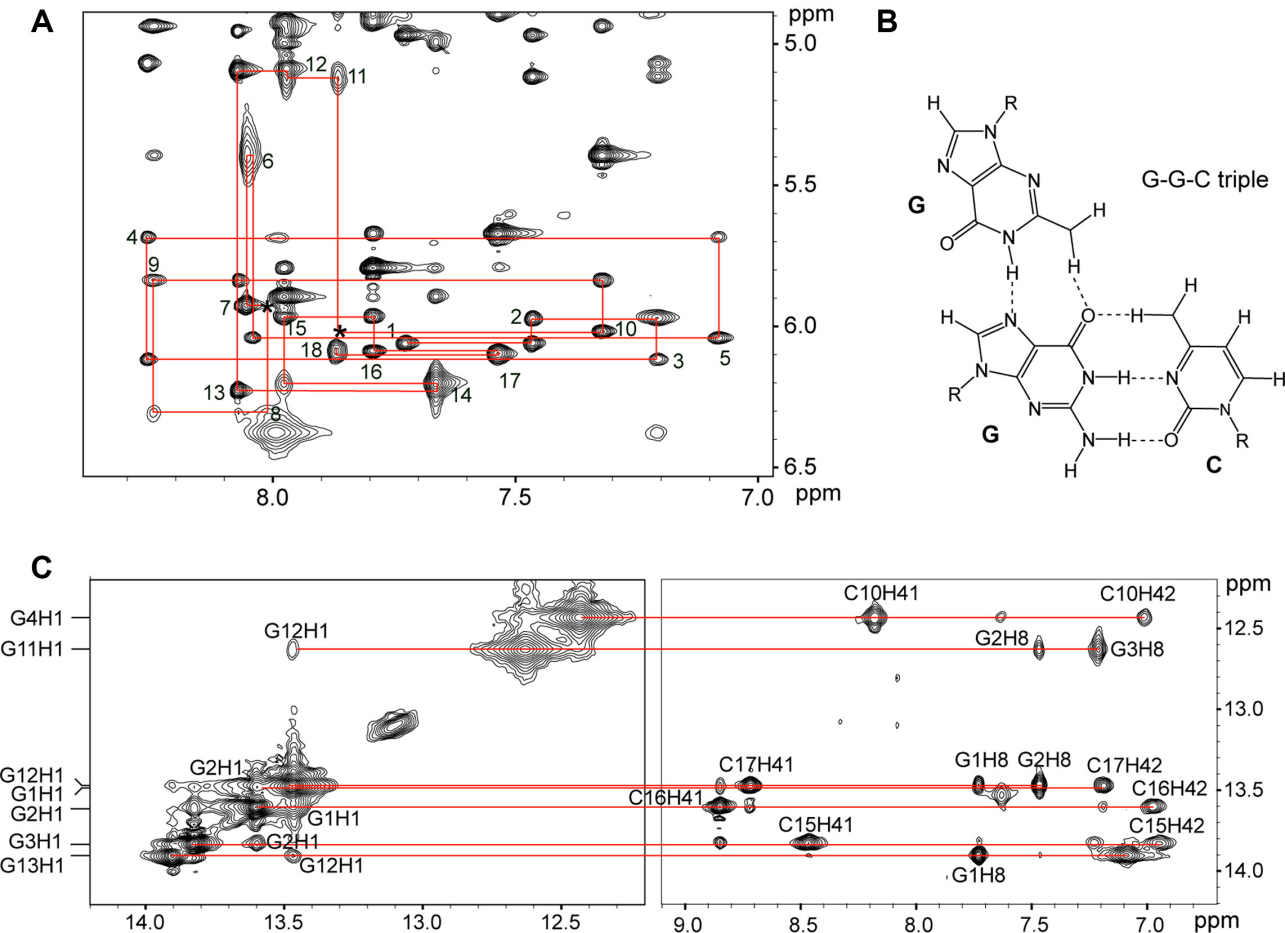
Spectra of the OBAwt OTA complex in phosphate buffer containing Mg^2+^. (**A**) NOESY spectra showing H8/H6-H1′ connectivity in D_2_O at 288 K (mixing time, 300 ms). Intraresidue H6/H8-H1′ NOE cross-peaks are labeled. Locations mark with an asterisk (*) represent unobserved cross peaks. (**B**) Schematic representation of G–G–C triple. (**C**) NOESY spectra showing H1 protons assignments H_2_O solvent at 288 K (mixing time, 100 ms).

To understand the discriminatory recognition of OBAwt, we collected a series of DQF-COSY, TOCSY and NOESY for the OBAwt─OTA complex and assigned the ^1^H resonances. Figure 2 shows the sequential connectivity through NOEs between H8/H6 and H1′ protons (Figure 2A) and imino H1 protons (Figure 2B). The H8/H6─H1′ NOE cross-peaks of the other nucleotides are observed, except for A_7_H1′─A_8_H8 and C_10_H1′─G_11_H8, which are probably >5 Å apart or because of local conformational dynamics (Figure 2A).

**Figure 3.**
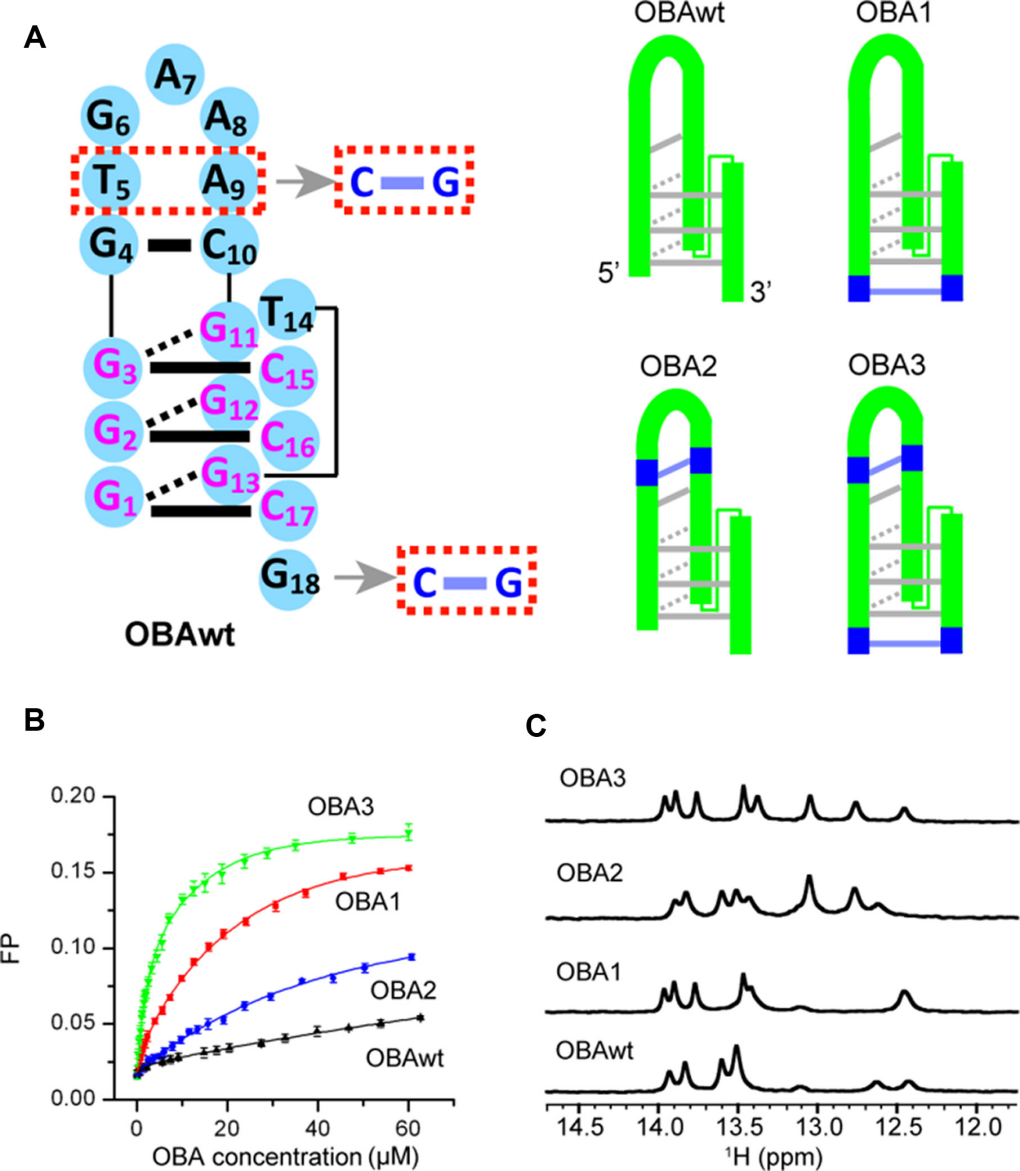
Secondary structures and function of the original OBA aptamer (OBAwt) and its variants (OBA1─3). (**A**) The sequence of OBAwt is shown on the left. The Watson-Crick and Hoogsteen hydrogen bonds are represented by solid- and dashed- bars, respectively. OBAwt and each variant are represented on the right. (**B**) Fluorescence polarization titration of OTA with OBA to test the effect of mutation on affinity (OBAwt, black; OBA1, red; OBA2, blue; OBA3, green). (**C**) Imino regions of ^1^H spectra of OBA with 2 equivalents of OTA in 10 mM Mg^2+^ at 288 K.

The imino protons of G1, G2, G3 and G4 show strong NOEs to the amino protons of cytosines C17, C16, C15 and C10, respectively, characteristic of G–C Watson–Crick pairing. The two amino protons were assigned by comparing the relative intensities of their respective NOE cross peaks with the guanine imino protons and with H5 protons of cytosine. The presence of strong NOEs from the imino protons of G11, G12 and G13 to the H8 protons of G3, G2 and G1, respectively, indicate a Hoogsteen-type interaction (Figure 2B). These results are in accord with the pairing interaction of a G–G–C triple (36), that is, three G–G–C triplets G11–G3–C15, G12–G2–C16 and G13–G1–C17 (Figure 3A), as demonstrated by the weak NOEs from G_11_H1─ G_12_H1 and G_12_H1─G_13_H1 and medium NOEs from G_11_H1─G_2_H8 and G_12_H1─G_1_H8.

For the GAA (G6─A8) fragment, both the NOEs among these bases and their chemical shifts, especially the unusual upfield-shifted resonances of A7 H4′, H5′ and H5″ (Supplementary Table S1), are consistent with the structure of the GAA loop (37,38), in which the NH_2_ and N3 atoms of the first G form hydrogen bonds with N7 and the NH_2_ of the third A. As reported elsewhere (38), direct evidence of G6–A8 pairing in our complex, that is hydrogen bond pairing, is not observed possibly due to chemical exchange. Nevertheless, the importance of the G6-NH_2_ group is confirmed by the loss of binding when replaced by hydrogen in inosine (Supplementary Figure S1 and Supplementary Table S2).

A schematic diagram of secondary structure of OBAwt based on the above base pairing information is shown in Figure 3a. The complex exhibits a hairpin structure with one G–C pair, three G–G–C triplets and one GAA loop. In addition, we observe NOEs between OTA and ^1^H of G4, C10, G11, G3, C15 and T14 (Supplementary Table S3), indicating that the binding pocket of OTA is likely composed of these nucleotides, and OTA is sandwiched between the two base planes of G4–C10 and G11–G3–C15. To identify the function of G–G–C triplets in recognizing OTA, the variant OBA4 with one fewer G in the G11–G13 segment was synthesized (Table 1). As shown in Supplementary Figure S2, OBA4 loses the ability to bind OTA, suggesting that the stability of triple helix is essential.

**Table 1.** Sequences and dissociation constants of aptamers

Name	Sequence	*K*_d_ (μM)
OBAwt	GGGGTGAAACGGGTCCCG	81 ±2
OBA1	CGGGGTGAAACGGGTCCCG	5.9 ±0.1
OBA2	GGGGCGAAGCGGGTCCCG	26.2±0.4
OBA3	CGGGGCGAAGCGGGTCCCG	1.4 ±0.1
OBA4	GGGGTGAAACG GTCCCG	NB^a^
OBA5	CCGGGGCGAAGCGGGTCCCGG	1.9 ±0.1
OBA6	GCGGGGCGAAGCGGGTCCCGC	2.4 ±0.1

^a^No binding.

### Structure-guided post-SELEX optimization of aptamer binding to OTA

The bases A9 and T5, next to GAA loop, do not form a pair of stable Watson-Crick hydrogen bonds, and the terminal G18 also lacks a complementary base in the OBAwt-OTA complex (Figure 3a), which likely reduces the affinity for OTA. Therefore, we changes T5 and A9 to C and G, respectively (OBA2) (Figure 3, Table 1), generating an aptamer with a GCGAAGC fragment that forms an extraordinarily stable hairpin (37). We then employed fluorescence polarization to determine the affinity for OTA. The *K*_d_ is 81 ± 2 μM for OBAwt, which is larger than the value (24 μM) determined by equilibrium dialysis (19), probably due to the difference of buffer and detection method. OBA2 shows a ∼3-fold improved affinity (26.2 ± 0.4 μM) compared to OBAwt. We also tried to add a C to the 5′-terminus to form G-C pair with G18 (OBA1) (Figure 3, Table 1). The fluorescence polarization binding assay indicates that OBA1 has a ∼13-fold improvement (5.9 ±0.1 μM), suggesting this G–C pair is important. More interestingly, when we mutated T5 and A9 to C and G, respectively, and added a C in the 5′-terminus (OBA3) (Figure 3, Table 1), the aptamer showed a >50-fold improvement in affinity (1.4 ± 0.1 μM), which suggests the stability of these local structures have a synergistic effect. We also tried to extend a G–C base pair on the terminus of OBA3 (OBA5 and OBA6) (Supplementary Figure S3, Table 1). The polarization data indicate a slightly decreased affinity, with a *K*_d_ 1.9 ±0.1 and 2.4 ± 0.1 μM for OBA5 and OBA6, respectively, indicating that the extension does not contribute affinity.

We used ^1^H NMR to observe discriminatory binding of these aptamers to OTA and OTB (Figure 3c and Supplementary Figure S4). Like OBAwt, OBA1–3 does not form well-defined structures in the absence of OTA, but form new structures upon addition of OTA. Moreover, the OBA3 aptamer with 2 equivalents of OTA gave rise to an improved spectrum compared to OBAwt, OBA1 and OBA2, indicating that OBA3─OTA is more stable than other complexes, in agreement with the results from fluorescence polarization. The OBA1–3 aptamers also retains high specificity; ^1^H spectra of OBA1–3 with 2 equivalents of OTB show that these aptamers weakly bind OTB (Supplementary Figure S4).

In summary, we designed a new aptamer with >50-folded improved affinity by analyzing OBAwt secondary structure, suggesting that our structure-guided post-SELEX approach can efficiently optimize aptamers, and serve as an alternative strategy for post-SELEX or Post-ExSELEX optimization (16,39).

### Three dimensional, high-resolution structure of OBA3─OTA

**Figure 4.**
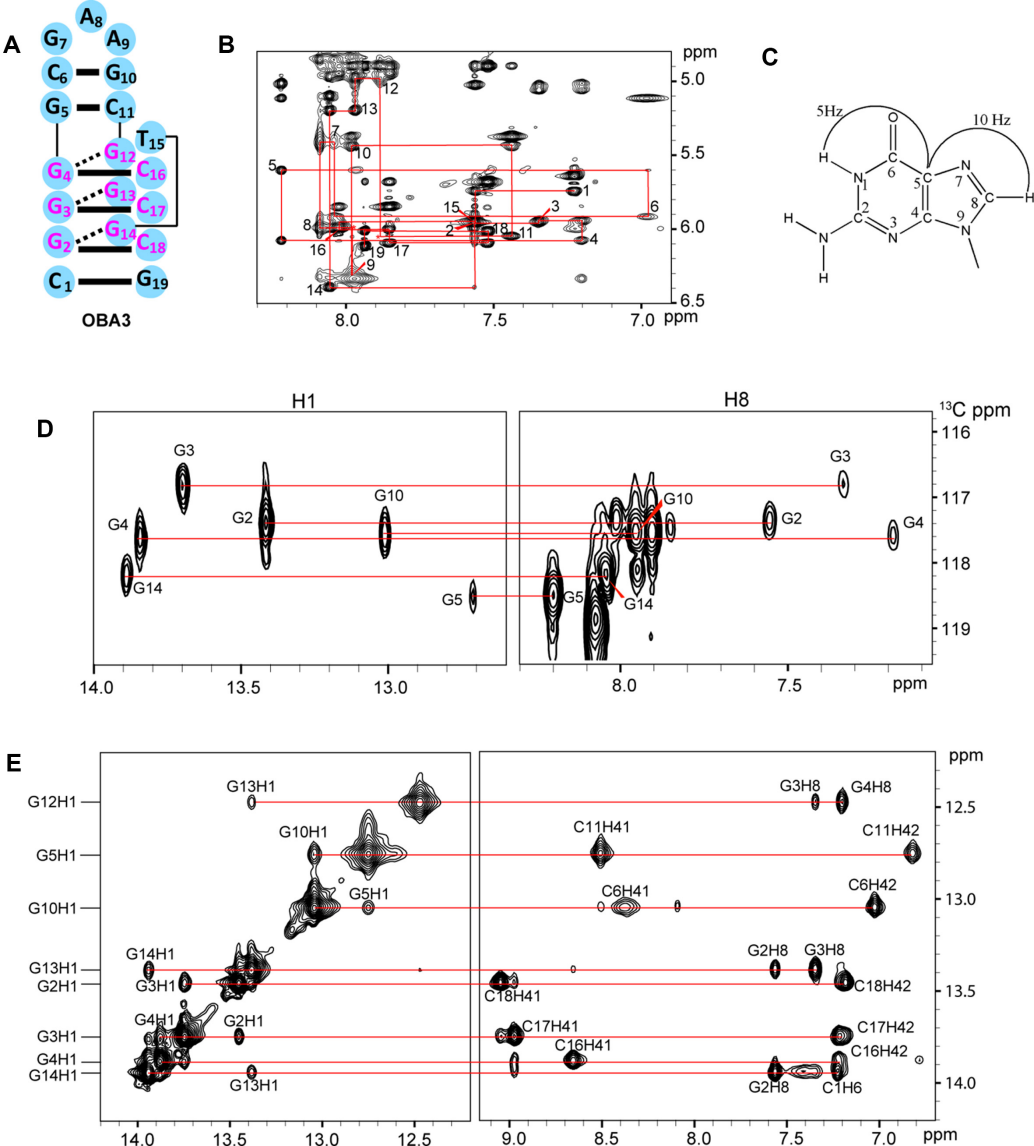
NMR spectra of the OBA3─OTA complex in phosphate buffer containing Mg^2+^. (**A**) The sequence of OBA3. The Watson-Crick and Hoogsteen hydrogen bonds are represented by solid- and dashed- bars, respectively. (**B**) NOESY spectra showing H8/H6-H1′ connectivity of OBA3 in D_2_O at 288K (mixing time, 300 ms). Intraresidue H6/H8-H1′ NOE cross-peaks are labeled with residue numbers. Peaks labeled (*) are not observed. (**C**) A schematic indicating long-range J-couplings between imino and H8 protons via ^13^C5 within the guanosine base. (**D**) H1 and H8 proton assignments by through-bond correlations between imino and H8 protons via ^13^C5 shown in (C) at natural abundance at 298 K. (**E**) NOESY spectra showing H1 protons assignments of OBA3 in H_2_O at 288 K (mixing time, 300 ms).

The superior spectral quality and improved affinity of OBA3, allowed us to determine the solution NMR structure of OBA3─OTA, and elucidate the structural basis for recognition of OTA, but not OTB. We recorded a series of 2D spectra and assigned the ^1^H protons signals (Supplementary Table S4). Figure 4 shows the correlation of imino H1 protons with amino/base protons (Figure 4E), the sequential connectivity through NOEs between H8/H6 and H1′ protons (Figure 4B) and the assignments of H1 and H8 protons by through-bond correlations between imino and H8 protons via ^13^C5 (Figure 4C and D).

As expected from our OBAwt data, NOESY spectra indicate that one G–C pair G5–C11, and three G–G–C triplets G12–G4–C16, G13–G3–C17 and G14–G2–C18 form in OBA3 aptamer (Figure 4). Unlike OBAwt, however, the presence of strong NOEs from imino protons of G10 to the amino protons of C6 in OBA3 indicate the formation of Watson–Crick pairing. In addition, the C1–G19 base pair forms in OBA3 (although, the imino proton of G19 is not observed possibly due to chemical exchange), because the two amino protons of cytosine C1 show different chemical shifts (8.05 and 7.33 ppm, respectively), and NOEs between the two amino protons and the amino protons of C18 are observed.

**Figure 5.**
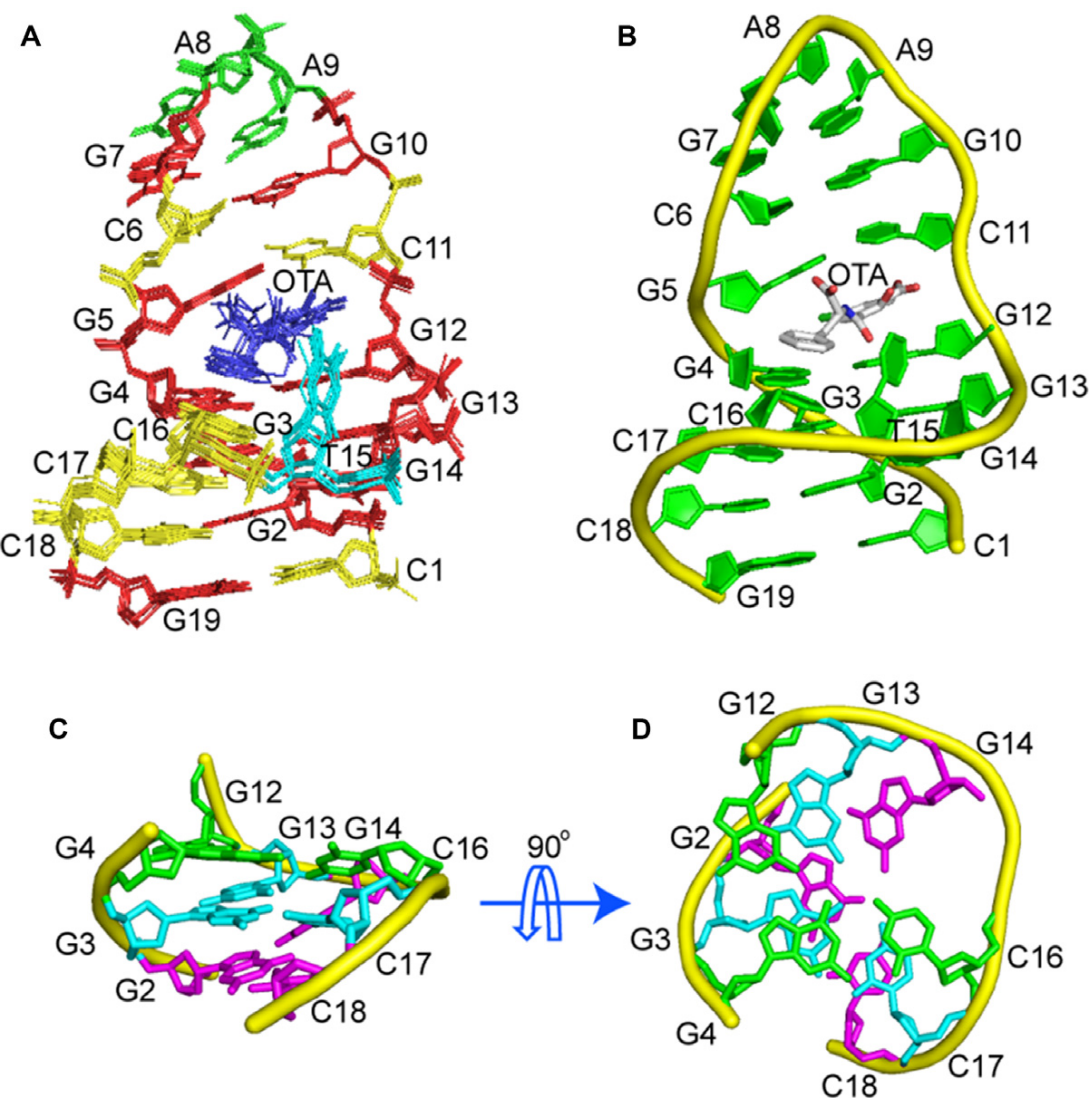
Structure of the OBA3─OTA complex. (**A**) Ten superimposed refined structures. (**B**) Cartoon representation of the lowest energy structure. (**C, D**) Triplex structure formed within OBA3─OTA complex. The G12:G4:C16 plane, the G13:G3:C17 plane and the G14:G2:C118 plane are colored green, cyan and magenta, respectively. Prepared using PyMOL (Version 0.99rc6, Schrodinger, LLC).

The structure of the OBA3─OTA complex was calculated using restraints, from NOEs, hydrogen bonds and dihedral angles obtained from NOESY and ^31^P─^1^H COSY spectra (Table 2, Supplementary Table S5) by using Xplor (29–31). Ten superimposed, lowest energy, refined structures and a cartoon view are shown in Figure 5A and B, respectively. The statistics of the structures are shown in Table 2. Among the structures, the average pairwise RMSD of all heavy atoms is 0.5 ± 0.2 Å for entire complex, and 0.4 ± 0.1 for entire complex without OTA, showing that the structures are well-defined.

**Table 2.** Statistics of the computed ten structures of OBA3─OTA complex

Distance restraints	
Intraresidue	238
Sequencial	110
Long-range	14
Intermolecular	44
Other restraints	
Hydrogen bond restraints	67
Sugar pucker restraints	38
Backbone dihedral angles	65
NOE violations	
Number (>0.2 Å)	0
RMSD of vilations (Å)	0.030 ± 0.000
Deviations from the ideal covalent geometry	
Bond lengths (Å)	0.002 ± 0.000
Bond angles (°)	0.485 ± 0.005
Impropers (°)	0.47 ± 0.03
Pairwise all heavy atoms RMSD values (Å)	
Entire complex	0.4 ± 0.1
Entire complex less OTA	0.4 ± 0.1

As shown in Figure 5, the upper part of OBA3 forms a stable hairpin via the GCGAAGC fragment, and the lower part is mainly a triple helix formed by three G-G-C triplets. GAA forms a mini-hairpin loop, in accordance with previous reports (37,38). The GAA loop has a compact structure stabilized by stacking interactions. Adenine A8 stacks on top of G7, and the sugar of A8 stacks on top of A9, explaining the unusual upfield-shifts of A8 H4′ (2.07 ppm), H5′ (2.98 ppm) and H5″ (3.35 ppm). The GAA loop has a sheared G-A pair with two hydrogen bonds, G7NH_2_–A9N_7_ and A9NH_2_–G7N_3._ Like OBAwt and the observation of Ulyanov (38), we did not obtain direct evidence for these two hydrogen bonds, possibly due to chemical exchange. However, the NOEs and chemical shifts within the GAA loop, especially the unusual upfield-shifted A8 H4′, H5′ and H5″ are consistent with the previous report, which strongly supports the structure in OBA3. Therefore, the two hydrogen bond restraints were used in structural calculation.

Also, like the observation of Ulyanov (38), the intensity of the intraresidue H1′-H8 cross-peak is much higher for A8 than for other residues, and the estimated distance is ∼3.3 Å based on the NOE cross-peak volumes from an NOESY spectrum with a 120 ms mixing time (as a reference, the cytosine base H5–H6 distance is 2.5 Å). A typical H1′-H8 distance is 3.8 and 2.5 Å for *anti-* and *syn-*conformations, respectively. The shorter distance suggests that A8 is mostly in the *anti*-conformation. This result is supported by the observation of strong NOEs between A8 H8 and G7 H2′/H2″ (*anti*-conformation) and weak NOEs between A8 H8 and A9 H3′/H4′/H5′/H5″ (*syn*-conformation). We used NOEs restraints from the *anti*-conformation.

### Binding pocket architecture

**Figure 6.**
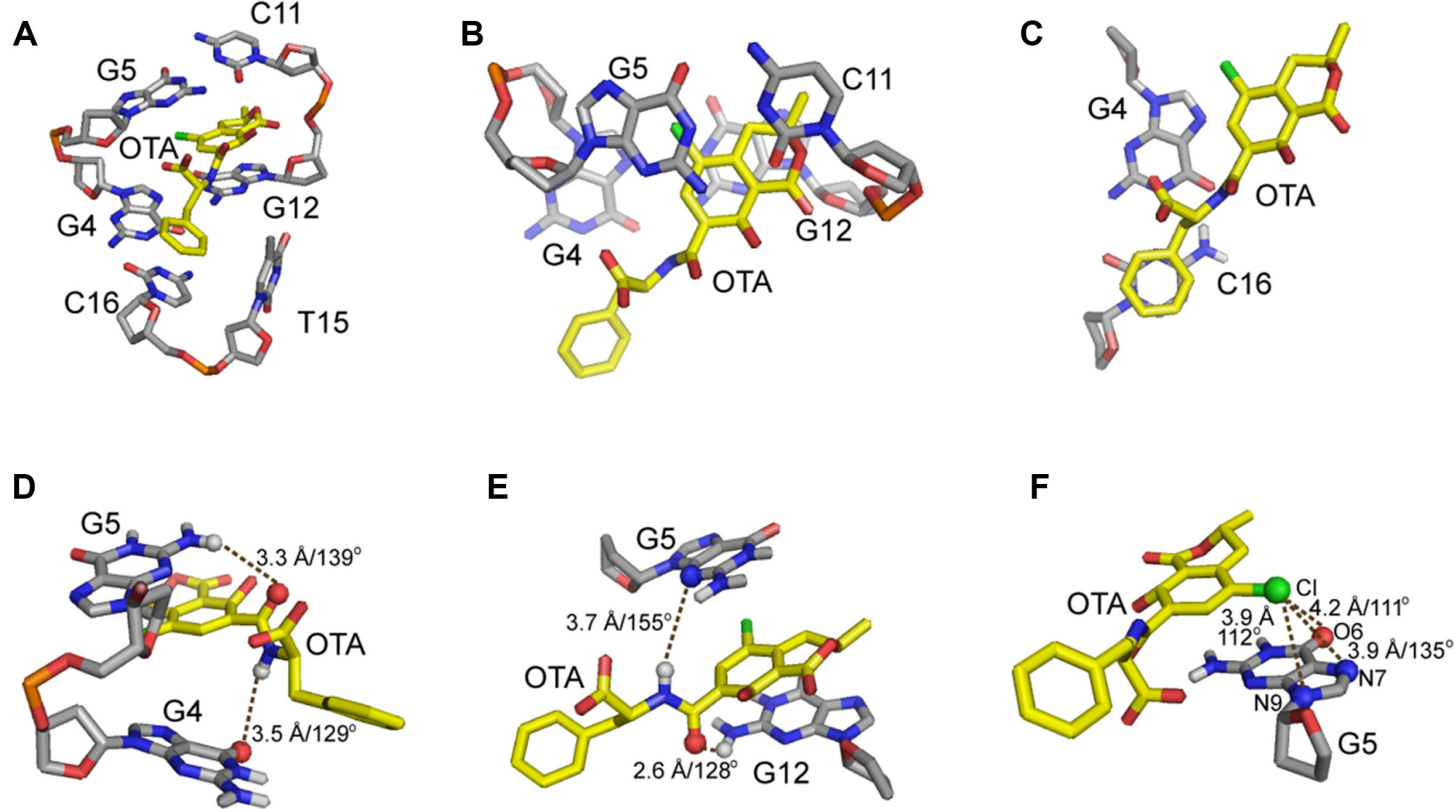
Expanded view of the OTA-binding site in the OBA3-OTA structure. (**A**) A stick view of the binding pocket. (**B**) Stacking of the isocoumarin ring of OTA between G5–C11 and G4–G12. (**C**) Stacking of the benzene ring of OTA on G4–C16. (**D**–**E**) Two models of intermolecular hydrogen-bonding between OTA and OBA3. (**F**) The potential hydrogen- and halogen-bonds are shown as dashes. Interaction lengths and angles are provided.

The residues involved in OTA binding in OBA3 DNA are shown in Figure 6. They include residues G4, G5, C11, G12, T15 and C16, which form a binding pocket to fit OTA. OTA is anchored by a hydrophobic interaction between T15 and the benzene ring of OTA, the hydrogen bonds between the amide group of OTA and residues G4, G5 and G12, the halogen bonds between the chlorine atom of OTA and G5, and the stacking of OTA between the G5–C11 base pair and G12–G4–C16 triple.

As shown in Figure 6, T15 does not pair or stack with other bases, but its location is well defined due to interactions with the benzene ring of OTA. Furthermore T15 occupies a *syn*-conformation to interact with the benzene ring of OTA via its methyl group, closing the binding pocket. The role of T15 in complex formation implies that its site-specific substitution by an unnatural nucleotide with a more hydrophobic base such as 7-(2-thienyl)imidazo[4,5-b]pyridine (Ds) (40–44) might further improve affinity.

The intermolecular hydrogen bond restraints between OTA and the aptamer were not used in structure calculation, because we did not observe the direct evidence in NOESY spectra. However, our 10 lowest energy structures show potential intermolecular hydrogen bonds between the amide group of OTA and the aptamer. Six of the ten conformers adopt the model shown in Figure 6D, with hydrogen bonds between the amide hydrogen of OTA and the O6 carbonyl oxygen of G4, as well as between the hydrogen from the amino group (NH_2_–2 group) of G5 and the carbonyl oxygen of the OTA amide. The other four conformers adopt the model in Figure 6E, in which hydrogen bonds form between the OTA amide hydrogen and the N3 atom of G5, and between the hydrogen from the amino group (NH_2_–2 group) of G12 and the carbonyl oxygen from the OTA amide.

A halogen bond is an interaction between a halogen and a Lewis base or another electron-rich moiety. The electron density donors are usually electronegative atoms such as oxygen, nitrogen, sulfur, aromatic rings or conjugated π-systems. Halogen-bonds are observed not only in protein-ligand complex but also in nucleic acid complexes (45). Halogen bonds are assigned using the criteria that the distance between the halogen and the oxygen, nitrogen, or phosphorus is less than 4.2 Å and the angle R-X··· O/N/P is more than 110° (where X is the halogen). According to these criteria, the G5 N7/N9/O6 atoms form halogen bonds to the chlorine of OTA (Figure 6F). The mean distances and angles are 3.9 Å/135°, 3.95 Å/112° and 4.19 Å/111°, respectively.

Three G–G–C triplets, G12–G4–C16, G13–G3–C17 and G14–G2–C18, form a continuous, stacked helix on complex formation. The triplets contribute important structural components to the binding pocket. They widen the minor groove, providing a platform large enough for both isocoumarin and the benzene rings of OTA to stack onto the G12–G4–C16 triple and allowing T15 to close the binding site by hydrophobic interaction. In addition, the stacking of OTA on G12–G4–C16 triple stabilizes the triple helix structure as observed in other triplex-binding systems (46–49).

Comparing our OTA–aptamer structure to the structures of other aptamer–ligand complexes (50–56) we note that intramolecular base triplets are also observed in AMP–DNA aptamer and argininamide-DNA aptamer complexes, and directly involve binding pocket formation and recognition of ligand, suggesting that mismatches or Hoogsteen hydrogen bonds play important roles in ligand recognition. The difference is that our three triplet alignment forming the pocket is continuous and along a single helix axis, an arrangement that had never been observed.

### Structural insights into discriminatory recognition

The only difference between OTA and OTB is the chlorine atom in the para-position of the phenol. Due to the effect of chlorine electronegativity, the p*K*_a_ of the phenolic hydroxyl (Ph-OH) is 7.1 for OTA (57), smaller than the value of 7.8 for OTB (58). Under our condition, pH 7.4, the Ph-OH proton has mostly dissociated for OTA, but not OTB. A titration on a mixture of OBA3 and OTB was performed to verify that this difference explains the discriminatory recognition (Supplementary Figure S5). The interactions (hydrogen bonds, stacking, hydrophobic interactions) between OTB and OBA3 should be the same as those for OTA, except for the absence of the halogen bonds. However, there is no significant change in aptamer binding to OTB as the pH increases, suggesting that dissociation of the Ph-OH proton is not the main reason for discriminatory recognition, rather the halogen bonds are likely responsible.

## DISCUSSION

### Watson-Crick pairs, base triple alignments and the OBAwt-OTA complex

The OTA–aptamer complex structure shows that the adaptive structural transition of is achieved, in part, through formation of Watson-Crick base pairs involving G1–C17, G2–C16, G3–C15, G4–C10 in OBAwt–OTA complex. These base pairs are of great importance; the OBAwt-OTA complex does not form when any of the Gs are replaced with inosine (one fewer hydrogen bond, Supplementary Figure S1, Supplementary Table S2).

We also observed three base triplets in the OBAwt-aptamer complex. G11 forms two hydrogen bonds through its NH and NH_2_–2 group with N7 and the O6 carbonyl of the G3–C15 Watson–Crick pair, respectively. G12 and G13 form hydrogen bonds with the G2–C16 and G1–C17 Watson-Crick pairs in the same manner. We observe the amino protons resonances of G12 and G13, but not G11, in NOESY spectra. The importance of G11 NH_2_–2 group, however, is confirmed by the loss of binding when it is replaced by hydrogen in inosine (Supplementary Figure S1, Supplementary Table S2). Similarly, we observed the roles of G12 and G13 bases NH_2_–2 groups. Replacing G13 with inosine prevents complex formation, but the aptamer where inosine replaces G12 still binds OTA. This observation suggests that the hydrogen bonds across the G11–G3 and G13–G1 base pairs are crucial to triple helix stability and complex formation yet one fewer hydrogen bond, across G12-G2, in the middle of triple helix is not a fatal blow to triple helix stability and ligand recognition.

### Halogen bonds and complex formation

H-bonding, stacking interactions, hydrophobic interactions, all play a role in OTA complex formation. Halogen bonds, however, make crucial contributions as, demonstrated by the weak binding of OBA3 to OTB and little or no binding of OTB to OBAwt. Although halogen-bonding is observed in nucleic acid complexes (45), to the best of our knowledge, no aptamers employing halogen-bonding in ligand recognition have been reported. Here we provide an example.

### Aptamer structure is important for affinity improvement

Our data show that introducing an extraordinarily stable mini-hairpin DNA and reinforcing the stem region with G-C pairs in OBAwt aptamer improve affinity, consistent with a previous report (39). The key to this strategy is knowing the structure of the complex, which provides the information required for optimization. Structural prediction tools such as Mfold may provide secondary structure information, but even then it can be difficult to predict the correct structure because aptamers can adopt unconventional base pairing in their binding pockets, as observed in existing aptamer–ligand complexes structures (51–53). As an example, commonly used software does not predict the correct secondary structure of the OBA aptamers studied here. Under these circumstances, experiment-based structural determination is crucial to the rational optimization of high affinity aptamers.

## CONCLUSION

Based on experimental structural information of an OTA aptamer, we improved affinity >50-fold, demonstrating that structure-guided post-SELEX is a useful alternative approach for post-SELEX or post-ExSELEX optimization. We also determined the first high-resolution NMR solution structure of an aptamer–OTA complex. The structure of OBA3─OTA complex reveals that halogen bonds between the chlorine of OTA and G5 are responsible for discriminating between OTA and OTB. Our study not only provides a detailed structural basis for understanding the molecular mechanism of discriminatory recognition, but also provides a structure-based rational design method for affinity improvement. The present research contributes to a deeper understanding of molecular recognition and will facilitate the development of new aptamer biosensors for biomedical research.

## DATA AVAILABILITY

The coordinates of the OBA3─OTA complex have been deposited in the Protein Data Bank (accession number: 6J2W).

## Supplementary Material

gkz336_Supplemental_File
