# Supplementary material for: Structure-guided post-SELEX optimization of an ochratoxin A aptamer
Source: Nucleic Acids Res. 2019 May 7;47(11):5963–72. doi: 10.1093/nar/gkz336 (PMC6582339; doi:10.1093/nar/gkz336)
Supplement: gkz336_Supplemental_File [file gkz336_supplemental_file.pdf]

Supplemental Information

Structure-guided post-SELEX optimization for an ochoratoxin A aptamer

Guohua Xu[1], Jiajing Zhao[1,2], Na Liu[1,2], Minghui Yang[1], Qiang Zhao[2,3], Conggang Li[1,2,]* and Maili Liu[1,2]

[1] Key Laboratory of Magnetic Resonance in Biological Systems, State Key Laboratory of Magnetic Resonance and Atomic and Molecular Physics, National Center for Magnetic Resonance in Wuhan, Wuhan National Laboratory for Optoelectronics, Wuhan Institute of Physics and Mathematics, Chinese Academy of Sciences, Wuhan, 430071, P. R. China.
[2] University of Chinese Academy of Sciences, Beijing, 100029, P.R. China.
[3] State Key Laboratory of Environmental Chemistry and Ecotoxicology, Research Center for Eco-Environmental Sciences, Chinese Academy of Sciences, Beijing, 100085, P. R. China.

* Corresponding Authors

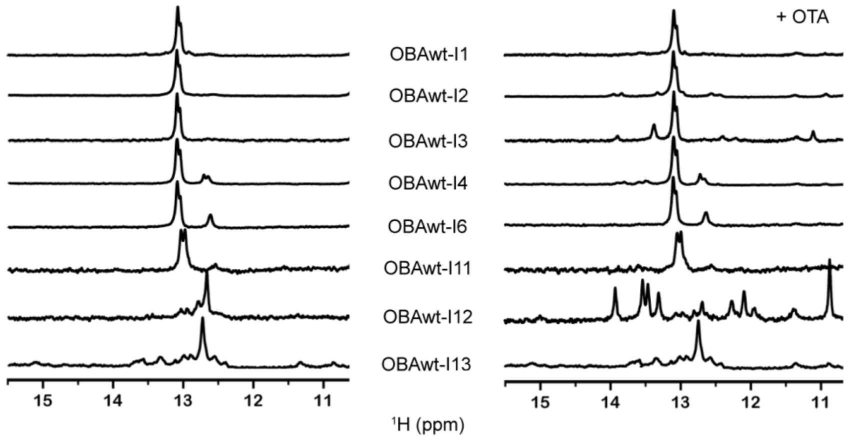

Figure S1. Imino regions of 1D ^{1}H NMR spectra of inosine-substituted OBAwt aptamers in phosphate solution containing Mg^{2+} at 288 K. ^{1}H NMR spectra of aptamer itself and with the addition of 2 equivalents of OTA are shown on the left and right, respectively.

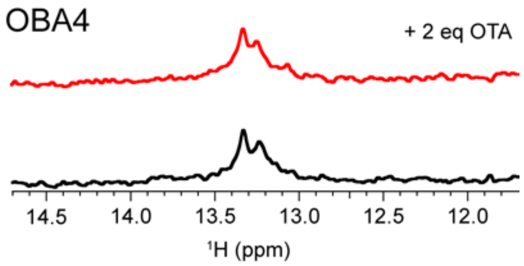

Figure S2. Imino regions of 1D ^{1}H NMR spectra of OBA variant OBA4 in phosphate solution containing Mg^{2+} at 288 K. ^{1}H NMR spectra of OBA itself (black) and with the addition of 2 equivalents of OTA (red), respectively.

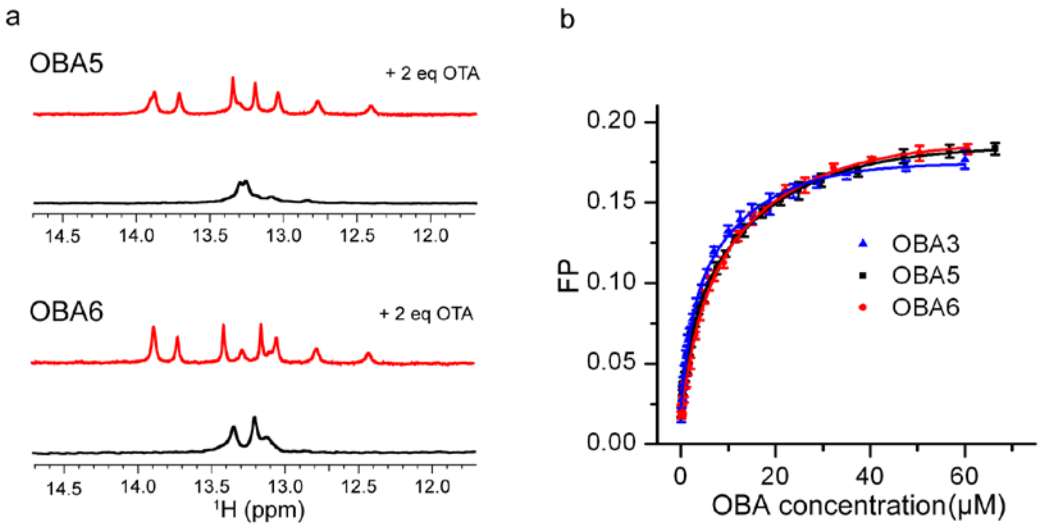

Figure S3. (a) Imino regions of 1D ^{1}H NMR spectra of OBA variants (OBA5 and OBA6) in phosphate solution containing Mg^{2+} at 288 K. ^{1}H NMR spectra of OBA itself and with the addition of 2 equivalents of OTA are colored black and red, respectively. (b) Fluorescence polarization titration of OTA with aptamers to test the effect of mutation on the binding affinity of the aptamer. The change of OTA fluorescence polarization is plotted as a function of aptamer concentration. The concentration of OTA is 0.5 μM.

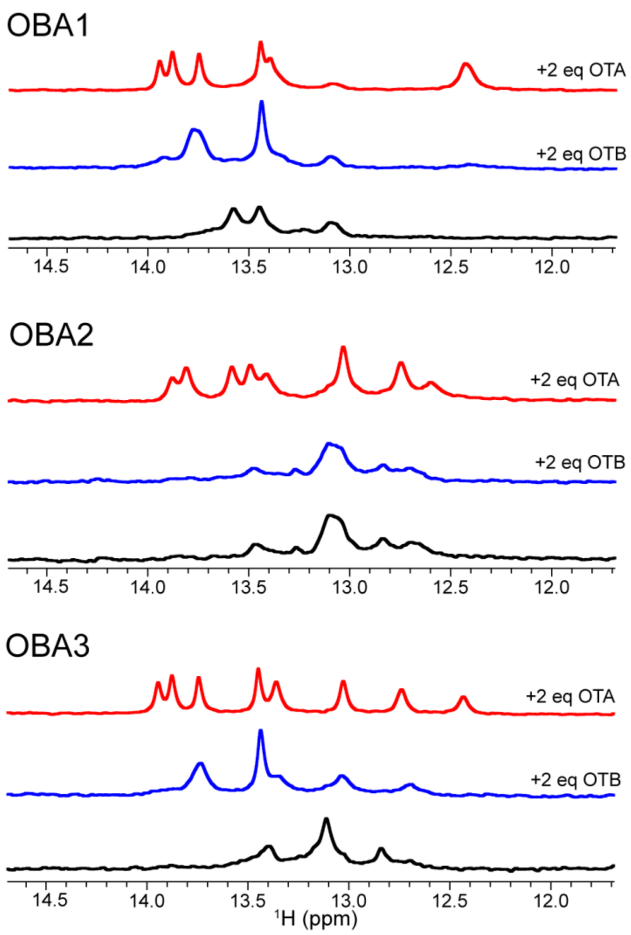

Figure S4. ^{1}H NMR spectra of OBA variants (OBA1–3) in phosphate solution containing Mg^{2+} at 288 K. ^{1}H NMR spectra of OBA itself, with the addition of 2 equivalents of OTA, 2 equivalents of OTB are colored black, red and blue, respectively.

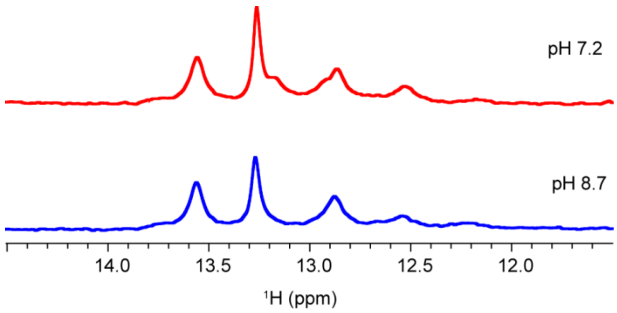

Figure S5. Imino region of 1D ^{1}H NMR spectra of OBA3 with 2 equivalents of OTB in phosphate solution containing Mg^{2+} at 288 K. ^{1}H spectra at pH 7.2 and 8.7 are colored blue and red, respectively.

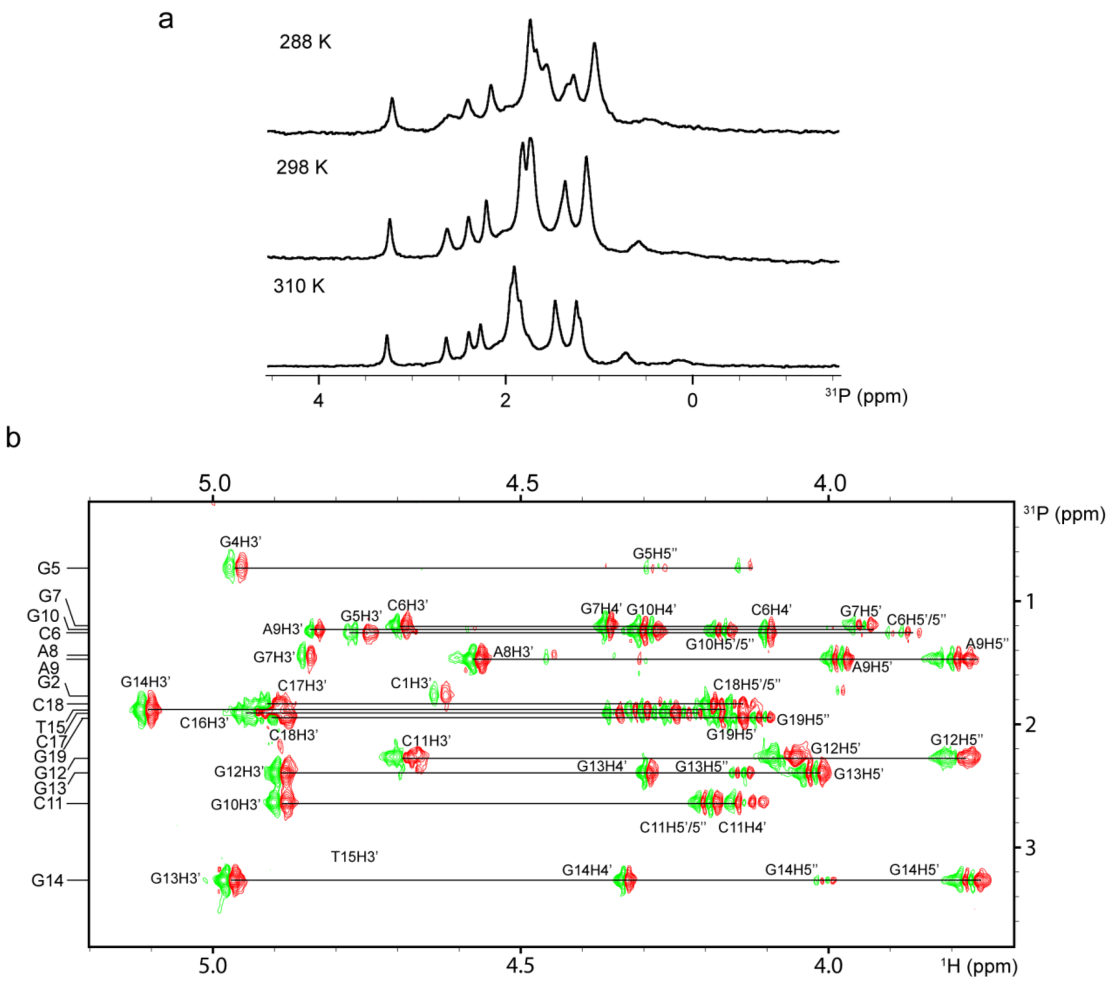

Figure S6. 1D ^{31}P and 2D heteronuclear ^{31}P–^{1}H Correlation Spectroscopy (COSY) of OBA3–OTA complex. (a) The 1D ^{31}P spectra of the complex at different temperature. The spectra were referenced to external 85% H_3PO_4. (b) COSY of the complex at 310 K.

Table S1. Proton chemical shift of OBAwt─OTA complex at pH 7.4,15 °C.

Residue	H1	H41/H21	H42/H22	H5/Me	H1’	H2’	H2”	H3’	H4’	H5’	H5”	H8/H6
G1	13.47				6.06	2.72	3.02	4.97	4.21	3.56	3.61	7.73
G2	13.60				5.97	2.68	2.92	5.11	4.39	4.25	4.21	7.46
G3	13.83				6.11	2.08	2.78	5.07	4.47	4.37	4.19	7.21
G4	12.43				5.68	1.98	2.42	4.74	4.14	4.18	4.40	8.26
T5				1.31	6.04	1.44	2.25	4.74	4.19	3.93	4.19	7.08
G6					5.39	2.65	2.51	4.86	4.36	3.94	4.03	8.04
A7					5.93	2.20	2.29	4.52	2.00	2.96	3.32	8.05
A8					6.31	2.97	2.97	4.85	4.35	3.98	3.80	8.02
A9					5.83	2.39	2.69	4.94	4.32	4.21	4.38	8.25
C10		8.17	7.00	5.39	6.02	2.34	2.48	4.75	4.09	4.11	4.20	7.32
G11	12.63				5.12	2.36	2.48	4.81	4.27	4.06	3.89	7.87
G12	13.47	6.41	6.41		5.09	2.57	2.74	4.96	4.30	4.02	4.12	7.97
G13	13.90	7.10	7.10		6.23	2.86	2.47	5.10	4.37	3.85	4.04	8.07
T14				1.90	6.20	2.56	2.64	4.99	4.35	4.21	4.30	7.67
C15		8.46	6.93	5.89	5.97	2.46	2.67	4.93	4.35	4.20	4.25	7.97
C16		8.85	6.99	5.79	6.09	2.24	2.56	4.89	4.25	4.16	4.20	7.79
C17		8.73	7.19	5.67	6.10	2.14	2.50	4.87	4.23	4.12	4.12	7.54
G18					6.08	2.49	2.38	4.66	4.10	4.19	4.19	7.87

Table S2. OBAwt aptamer and its inosine-substituted variants sequences.

Name	Sequence
OBAwt	GGGGTGAAACGGGTCCCG
OBAwt-I1	**I** GGGTGAAACGGGTCCCG
OBAwt-I2	G **I** GGTGAAACGGGTCCCG
OBAwt-I3	GG **I** GTGAAACGGGTCCCG
OBAwt-I4	GGG **I** TGAAACGGGTCCCG
OBAwt-I6	GGGGT **I** AAACGGGTCCCG
OBAwt-I11	GGGGTGAAAC **I** GGTCCCG
OBAwt-I12	GGGGTGAAACG **I** GTCCCG
OBAwt-I13	GGGGTGAAACGG **I** TCCCG

Table S3. Intermolecular NOEs between OTA and OBAwt aptamer protons in OBAwt─OTA complex.

OTA protons	OBAwt aptamer protons
H3	G11-H8
H9	G3-H8, H2’/2’’<br>G4-H1’
H21	C10-H6, H5, H1’, H2’’<br>G11-H8, H5’/5’’
H16/20	T14- Me<br>C15-H5
H17/19	T14- Me<br>C15- H5, H1’

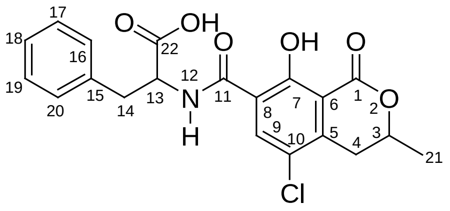

OTA structure

Table S4. Proton chemical shift of OBA3─OTA complex at pH 7.4,15 °C.

Residue	H1	H41/H21	H42/H22	H5/Me	H1’	H2’	H2’’	H3’	H4’	H5’	H5’’	H8/H6
C1		8.05	7.33	5.64	5.74	1.83	2.24	4.63	4.00	3.37	3.45	7.23
G2	13.45				5.95	2.60	2.93	5.03	4.28	3.98	4.00	7.56
G3	13.75				5.94	2.66	2.90	5.06	4.40	4.30	4.27	7.35
G4	13.88				6.08	1.93	2.66	5.01	4.46	4.34	4.16	7.20
G5	12.75				5.60	2.08	2.40	4.73	4.13	4.15	4.30	8.22
C6		8.37	7.02	5.12	5.92	1.41	2.19	4.70	4.12	3.87	3.88	6.98
G7					5.40	2.62	2.50	4.85	4.36	3.96	4.00	8.04
A8					5.99	2.25	2.29	4.56	2.07	2.98	3.35	8.08
A9					6.32	2.91	2.91	4.84	4.33	3.98	3.79	7.98
G10	13.04				5.43	2.52	2.52	4.90	4.32	4.17	4.17	7.98
C11		8.51	6.82	5.38	6.05	2.48	2.48	4.64	4.14	4.20	4.20	7.44
G12	12.47				4.97	2.53	2.58	4.89	4.28	4.07	3.82	7.89
G13	13.38	6.60	6.60		5.20	2.57	2.75	4.97	4.29	4.01	4.14	7.97
G14	13.94	7.40	7.40		6.39	2.89	2.50	5.10	4.32	3.71	3.97	8.06
T15				1.91	5.95	2.51	2.68	4.89	4.29	4.15	4.15	7.56
C16		8.66	6.62	5.99	6.00	2.56	2.69	4.95	4.35	4.24	4.29	8.02
C17		8.97	7.21	5.85	6.10	2.32	2.61	4.91	4.27	4.18	4.20	7.85
C18		9.05	7.18	5.68	6.01	2.07	2.59	4.90	4.20	4.16	4.16	7.52
G19					6.12	2.54	2.34	4.68	4.15	4.10	4.17	7.94

Table S5. Intermolecular NOEs between OTA and OBA3 aptamer protons in OBA3—OTA complex.

OTA protons	OBA3 aptamer protons
H3	G12-H8
H4	G5-H1<br>C11-H41
H9	G4-H8, H2’/2’’<br>G5-H8, H1’
H14	G4-H1<br>C16-H41
H21	C11-H6, H5, H41/42, H1’, H2’’, H4’, H5’/5’’<br>G12-H8
H16/20	G4-H1<br>T15-H6, H1’, Me<br>C16-H6, H5
H17/19	G4-H1<br>T15-H6, H1’, H2’/2’’, Me<br>C16-H6, H5, H1’
H18	T15-H6, H2’/2’’<br>C16-H6, H5

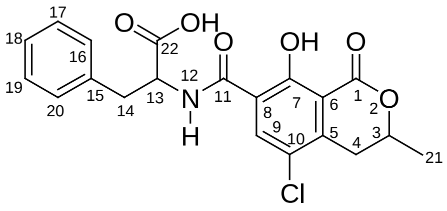

OTA structure
